# Comparative overview of the effects of aerobic and resistance exercise on anxiety-like behavior, cognitive flexibility, and hippocampal synaptic plasticity parameters in healthy rats

**DOI:** 10.1590/1414-431X20209816

**Published:** 2020-10-09

**Authors:** E. Segabinazi, N.F. Gasperini, A.M. Faustino, R. Centeno, A.S. dos Santos, W. de Almeida, L.P. Bronauth, S. Marcuzzo, L.O. Pereira

**Affiliations:** 1 Programa de Pós-Graduação em Neurociências, Instituto de Ciências Básicas da Saúde, Universidade Federal do Rio Grande do Sul, Porto Alegre, RS, Brasil; 2 Laboratório de Histofisiologia Comparada, Departamento de Ciências Morfológicas, Instituto de Ciências Básicas da Saúde, Universidade Federal do Rio Grande do Sul, Porto Alegre, RS, Brasil

**Keywords:** Strength exercise, Endurance training, Physical activity, Executive function, Emotion, Memory

## Abstract

Clinical studies show that physical exercise has anxiolytic and pro-cognitive properties for both healthy individuals and psychiatric patients. Most of these data refer to the effects of aerobic exercise. However, other modalities such as resistance exercise deserve more attention because they may also modulate brain function. This study aimed to compare the effects of an aerobic exercise protocol on a treadmill and a resistance exercise protocol on a ladder apparatus on anxiety-like behavior, cognitive flexibility, and neuroplasticity parameters in healthy animals. Adult male Wistar rats were divided into three groups: sedentary control, aerobic training, and resistance training. Subsequently, they were evaluated in the elevated plus-maze (EPM), light-dark box, and modified hole board (mHB) tests. The expressions of synaptophysin and postsynaptic plasticity protein 95 in the dorsal and ventral hippocampus were analyzed by immunofluorescence. The results demonstrated an anxiolytic effect promoted by exercise in the EPM, particularly in the animals submitted to aerobic training, and a mild pro-learning effect of both exercise modalities was observed in the mHB test. All groups showed similar outcomes in the other evaluations. Therefore, the exercise modalities investigated in the present study did not provide considerable modifications to such aspects of the emotional/cognitive functions and neuroplasticity under physiological contexts. Perhaps the two types of exercise acted in neurobiological pathways not analyzed in this study, or the effects may emerge under pathological contexts. These hypotheses should be tested in future studies.

## Introduction

The effects of aerobic exercise on neuroplasticity have been extensively studied in humans, including improvement of cognition/mood and hippocampal volume increase in both physiological and pathological contexts ([Bibr B01] [Bibr B02] [Bibr B03] [Bibr B04] 15). These positive effects were also observed in animal studies; anxiolytic effects of aerobic exercise have been widely demonstrated in standard tests to evaluate anxiety-like behavior in rodents, such as elevated plus-maze (EPM) and light-dark box (LDB) tests ([Bibr B06] [Bibr B07] 68). Also, it has been reported that aerobic exercise facilitates cognitive flexibility as well as other executive functions, such as inhibitory control and working memory (9).

Some molecular and cellular mechanisms are suggested for these behavioral phenotypes induced by aerobic exercise, such as an increase of growth factors and higher expression of synaptic plasticity markers, as synaptophysin (SYP) and postsynaptic density protein-95 (PSD-95) in the hippocampus (10,11). Treadmill running protocols have been extensively adopted in rodent studies of aerobic training effects because they allow precise evaluation of variables like frequency, intensity, and duration of a session, which is not possible in wheel running protocols. Also, the intensity of treadmill running is easier to measure compared to swimming, for which there is a lack of graded workload protocols, as reviewed by Seo et al. (12).

Resistance exercise has also been recommended by the World Health Organization for children and young people (13). In Brazil, resistance training is the third most prevalent exercise modality in young adult men (14). However, studies investigating its effects on neuroplasticity and behavior are recent and still scarce, principally if compared to aerobic exercise. The present literature demonstrates strong evidence that resistance exercise enhances cognitive functions and decreases anxiety in healthy individuals and in those with mental illness ([Bibr B15] [Bibr B16] 1517). In pre-clinical studies, resistance training improved the cognition of naive animals, and in Alzheimer’s disease and chronic stress models ([Bibr B18] [Bibr B19] 1820). However, effects on anxiety-like behavior of rodents have not yet been investigated. Some of these data were obtained from ladder-climbing resistance exercise model, which allows the definition of some parameters also used in aerobic exercise on a treadmill, such as frequency and intensity, and the comparison between the modalities. In addition, the ladder-climbing model is considered voluntary and does not use aversive stimuli like other resistance exercise protocols, which could have an adverse effect, as reviewed by Seo et al. (12).

Most preclinical and clinical studies that investigate exercise effects on cognitive performance and emotion are devoted to the aerobic modality, with the effects of other protocols such as resistance exercise less explored. In addition, the adherence to resistance training is higher among the physically active population in Brazil and its practice is recommended worldwide, so it is imperative that more studies explore the neurobiology of resistance exercise.

This study proposed a comparative overview on the effects of these two types of exercise - aerobic and resistance - on the anxiety-like behaviors of healthy animals. The modified hole board (mHB) test was also adopted with the aim of expanding our observation on cognitive processes in rats; through this task it is possible to evaluate cognitive function, including learning ability and cognitive flexibility (21). Additionally, we analyzed the plasticity markers in the dorsal and ventral hippocampus separately, because the former is related to the cognitive performance and the latter region, with the anxiety-like behavior ([Bibr B22] [Bibr B23] 2224).

## Material and Methods

### Animals

Sixty-one male Wistar rats (90 days old) were obtained from the local breeding colony (CREAL/ICBS, Universidade Federal do Rio Grande do Sul, Brazil). These animals were allocated in standard Plexiglas home cages (410×340×178 mm), 2 rats per cage, and kept in an environment with 20±2°C, 12-h light/dark cycle with lights on at 7:00 am, and food and water available *ad libitum*. All procedures were previously approved by the Ethical Committee at the Universidade Federal do Rio Grande do Sul (#32461) and were performed in accordance with the Guide for the Care and Use of Laboratory Animals adopted by the National Institutes of Health (USA) and the Arouca Law (Law No. 11.794/2008). Animals were randomly distributed among three groups: sedentary control (CT), aerobic exercise protocol on the treadmill (AE), and resistance exercise protocol on the ladder apparatus (RES). The exercise protocols were performed between 13:00 and 17:00 and the behavioral tests took place between 9:00 and 18:00. The sedentary rats were handled during the exercise period.

### Exercise paradigms

#### Aerobic protocol on a treadmill

**Figure 1:**
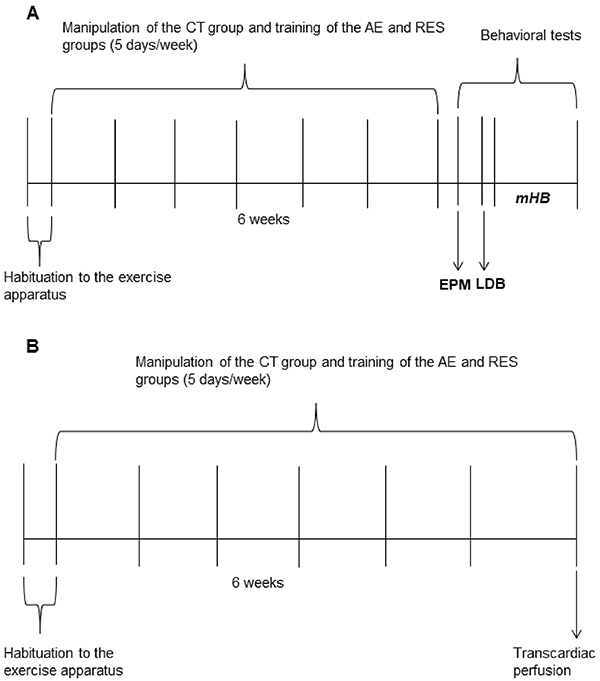
Timeline of the experimental procedures. At the end of the last exercise session, one cohort of animals was used for the behavioral tests (**A**) and the other for the immunofluorescence technique (**B**). CT: sedentary control; AE: aerobic exercise on the treadmill; RES: resistance exercise on the ladder apparatus; EPM: elevated-plus maze; LDB: light-dark box test; mHB: modified hole board test.

The animals were exercised on a horizontal motorized treadmill adapted to rodents, in separate Plexiglas lanes (INBRAMED TK 01, Brazil) during six weeks (Figure 1), five consecutive days in each week, 20 min/day at the intensity of 60% of maximal running speed (MRS) (adapted from Confortim et al. (25). Habituation to the treadmill before the beginning of exercise and MRS assessment were performed as described by Confortim et al. (25). MRS was measured every four exercise sessions in order to update the training speed when the animals' physical capacity improved. No aversive stimulus was used. When an animal refused to run even though stimulated with gentle touches, it was discarded from the sample, as occurred with three animals.

#### Resistance protocol on a ladder apparatus

Firstly, the rats of the RES group were habituated to the resistance training apparatus that consisted of an 80° inclined vertical ladder (110 cm high×18 cm wide, with 2-cm grid steps). At the top of the ladder, there was a dark box (20×20×20 cm), which was a stimulus to the rat climbing the ladder, because rats are attracted by the safety that closed and dark places represent against a possible predator. This box also served as a resting place until the next climb (10,26). Resistance exercise was performed on five consecutive days/week for six weeks (Figure 1). Each daily session was composed by eight series (climbs) of 8-12 repetitions (climbing movements) always carrying a load equivalent to 60% of the rats' maximal carrying capacity (MCC). The animals could rest during 60 s between climbs. The MCC was reevaluated every four exercise sessions to update the training load. The habituation and MCC protocol was performed as described by Novaes Gomes et al. (26). Two animals were excluded from the study because they refused to climb the vertical ladder.

At the end of exercise protocols, 46 animals were used exclusively for behavioral tests (Figure 1A). Another 15 rats were used for the immunofluorescence assay without undergoing behavioral tasks (Figure 1B).

### Anxiety-like behavior analysis

#### Elevated plus-maze

At the end of the exercise protocols, the anxiety-like behavior of animals was analyzed in the EPM test (Figure 1A) as described by Pereira et al. (27). Briefly, every rat was positioned individually in the central zone facing a closed arm and it could explore the apparatus during 5 min. The anxiety-like behavior was verified by the measurement of spatial-temporal variables, such as time spent in the open arms (s), and number of open and closed arms entries. The parameters are reported as percent of time spent in the open arms and percent of open arm entries. Furthermore, the number of risk assessments was also counted as an ethological variable. Risk assessment behavior was determined in this study as the movement in which the animal explores with the forepaws and head the open arm with a stretched posture. Each group was formed by 11-13 rats. The apparatus was illuminated only with a 15-w fluorescent light that was suspended 100 cm above the maze's central square to avoid shadow formation.

#### Light-dark box test

In the following day to the EPM test, the animals were exposed to the LDB paradigm (Figure 1A) as described by Sasaki et al. (28). The box was placed in a dimly lit environment with a single fluorescent lamp (15 w) centered on the white part of the box. Each animal was placed individually in the white chamber with its back to the dark compartment and left to explore the apparatus for 5 min. The wall that separates the two compartments has a small opening (10×10 cm) to allow the animal to pass from the light to the dark chamber. The time spent in the light compartment (s) was used to assess anxiety-like behavior. Each group was formed by 11-13 rats.

### Cognitive function

#### Modified hole board

The protocol of this study was adapted from Ohl et al. (21). This behavioral task started the following day the LDB paradigm (Figure 1A). This test runs in an open field apparatus (60×60×30 cm) in which are positioned nine bowls in the central area in such way that the animal can transit among the bowls and at the edges of the apparatus. Initially, three of these bowls were lined with cloth and filled with sawdust. Each covered bowl had a reward portion (1/4 of a Froot Loop ring) hidden under the sawdust (baited bowls). In the other uncovered bowls, there was only sawdust, without reward (non-baited bowls). This configuration was kept during the four days of the training phase (T1-T4, where T1 was the first training day and so on), and the animal had to learn that the cloth covering of the pots was the criteria for finding the reward. The rats underwent four trials daily. In each trial, they were placed individually in the arena and had a maximum time of 5 min to find the three food rewards. If the rat did not find the rewards in this time, it was gently guided to the baited bowls to eat the reward during the training phase. After each trial, the apparatus was cleaned with 20% ethanol used to remove odors.

In order to rule out the possibility of the animal finding the reward due to spatial memory, the position of the bowls was scrambled on the fifth day of test, setting a location shift phase. On the sixth day, a reversal phase was performed with the purpose of analyzing the cognitive flexibility of animals. For this, the reward was put in three uncovered bowls (baited but not cued) and the six covered bowls were cued but not baited. Moreover, the spatial distribution of the bowls was scrambled again. The following variables were analyzed in all phases of the test: 1) latency (s) to find the first and all three food rewards and 2) number of omission errors (baited bowls that were not visited).

A visit was considered when the animal smelled or dug into the pot with its paws or snout. All pots were impregnated with vanilla essence to stimulate rats to explore them. Froot Loops pieces were crushed and put in the bottom of the non-baited bowls every day before the beginning of the test. Two days before the training phase, the animals were familiarized with the Froot Loops and the bowls in their home cage. Both bowls contained clean sawdust and Froot Loops pieces from top to bottom for the animal to learn to dig. On the following day, each rat was habituated for 10 min in the mHB arena containing 5 bowls covered and 4 uncovered. On this day, there were rewards at the top and the bottom of all bowls. Each group was formed by 14-16 rats.

### Euthanasia and sample collection

One day after the last exercise session, 4-6 rats/group were anesthetized with an association of ketamine (90 mg/kg, Syntec, Brazil) and xylazine (10 mg/kg, Syntec) via *ip*. Then, they underwent transcardiac perfusion with 0.9% saline solution, and, posteriorly, with 4% paraformaldehyde diluted in 0.1 M phosphate buffer (PB; pH 7.4, Synth). The brains were removed from the skull, post-fixed in the previous fixative solution, and cryoprotected in 15 and 30% sucrose (Dinâmica, Brazil) solutions diluted in phosphate buffer saline (PBS). The samples were frozen in isopentane, cooled in liquid nitrogen, and stored at -80°C until to processing (adapted from Nicola et al. (29)). The dorsal (-2.56 to -4.52 mm from bregma) and ventral (-4.80 to -6.0 mm from bregma) hippocampus were sectioned in 30-μm thick coronal slices 180 μm apart at -20°C in a cryostat (Thermo Scientific, Germany) (30).

### Immunofluorescence for SYP and PSD-95

Immunofluorescence for each plasticity marker and region was performed separately. Initially, the sections were washed with PBS (pH 7.4), permeabilized with PBS containing 0.4% Triton X-100 (PBS-Tx), and blocked with 3% bovine serum albumin (BSA; Sigma Aldrich, USA) in PBS-Tx for 30 min. Then, the slices were incubated for 48 h, at 4°C, with the primary monoclonal antibodies: mouse anti-SYP (1:250, Sigma Aldrich) or mouse anti-PSD-95 (1:500, Millipore, USA), both diluted in 3% BSA. The samples were then washed again in PBS-Tx and incubated for 2 h with the secondary antibody goat anti-mouse Alexa Fluor 555 (1:500, Molecular Probes, Invitrogen, USA) diluted in PBS-Tx at room temperature. Lastly, the sections were washed in PBS, covered with aqueous mounting medium (Fluoromount, Sigma Aldrich), and coverslipped (adapted from Nicola et al. (29)).

### Quantification of hippocampal SYP and PSD-95 expression

Photomicrographs of dorsal and ventral *cornus ammonis* 1 (CA1) region from both hemispheres with a magnification of 400× were obtained with a fluorescence microscope (Nikon Eclipse E-600, Japan) coupled to a digital camera. The fluorescent labeling to SYP and PSD-95 in each image was evaluated from the mean of the integrated density of three areas of interest (AOIs) distributed in the *stratum radiatum*, each AOI measured 236,897,203 μm^2^, using ImageJ v. 1.80_112 software (IBM,USA) (adapted from Nicola et al. (29)). In each staining, 10-16 images from six slices per rat were analyzed. Data are reported as relative density (% of sedentary).

### Statistical analysis

The latency to find the first and all three food rewards and the number of omission errors during the training phase of mHB test were evaluated by one-way repeated measures ANOVA followed by the Tukey's *post-hoc* test. The other variables were analyzed by one-way ANOVA and Tukey's *post-hoc* test, when necessary. The level of significance was set at ≤0.05. Graphs were plotted using Graph Pad Prism 5 (USA). Data are reported as means±SE.

## Results

### Behavioral evaluation

**Figure 2:**
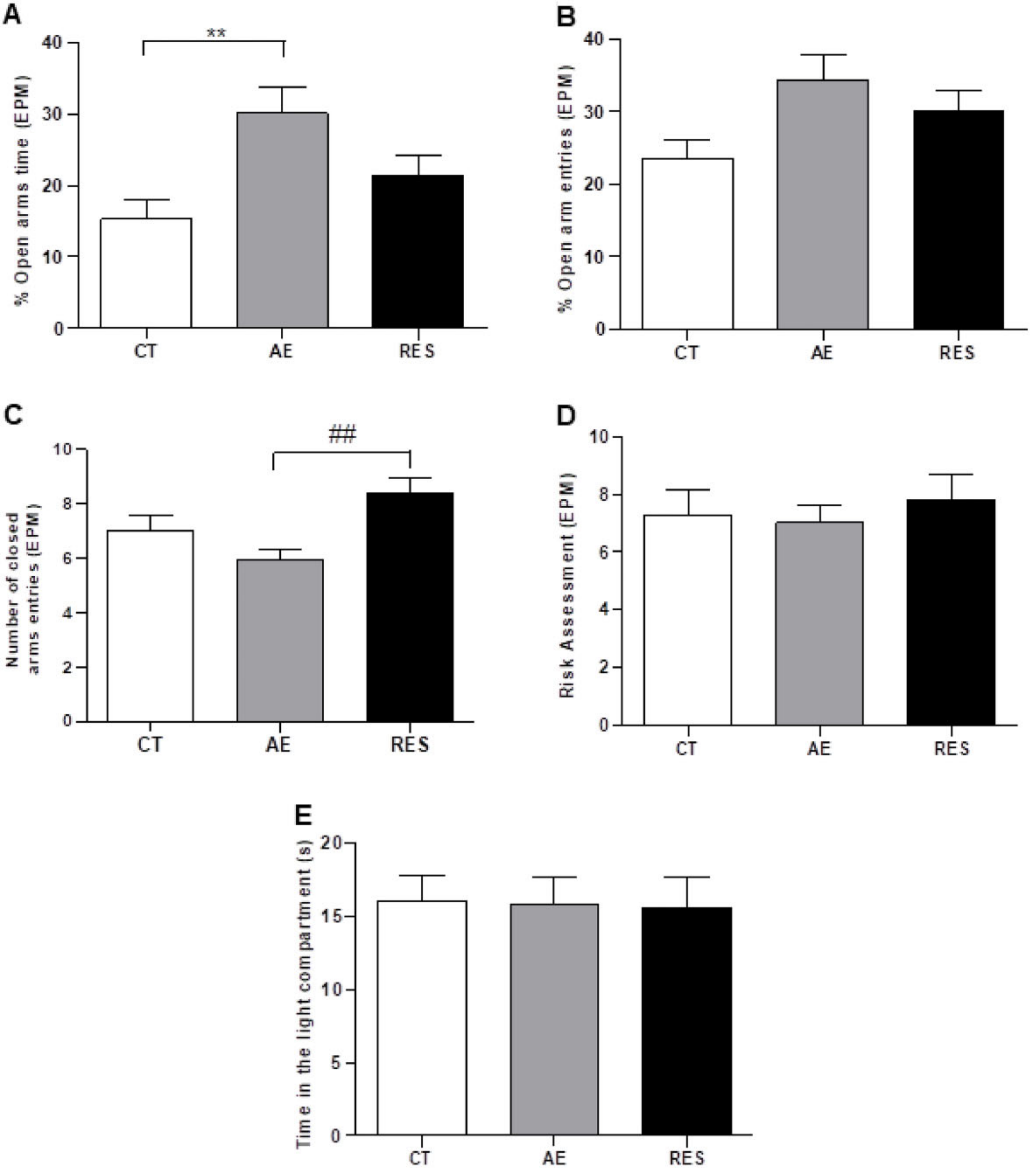
**2.** Anxiety-like behavior of rats in the elevated plus-maze (EPM) and light-dark box paradigms. **A**, Percentage of time spent in the open arms. **B**, Percentage of open arms entries. **C**, Number of closed arms entries. **D**, Number of risk assessments. **E**, Time spent in the light compartment. Data are reported as means±SE (n=11-13 per group). **P<0.01, ^##^P<0.01, one-way ANOVA followed by Tukey's *post hoc*. CT: sedentary control; AE: aerobic exercise on the treadmill; RES: resistance exercise on the ladder apparatus.

The animals that exercised on the treadmill presented a higher percentage of time spent in the open arms than the sedentary controls (F (2,33)=5.23, P=0.01; Figure 2A). A tendency for exercise impact on the percentage of open arms entries (F (2,33)=2.96, P=0.06; Figure 2B) was also found. These results indicated an anxiolytic effect of aerobic training.

Exercise also had a significant impact on the number of closed arms entries (F (2,33)=6.99, P<0.01) and the *post hoc* test indicated that the RES group increased closed arms entries compared to the AE group (Figure 2C). No difference was observed from the CT group. This suggests that the RES group had increased general exploration behavior of the EPM only compared to the AE group. Considering risk assessment behavior, no difference was detected (Figure 2D).

The time spent in the light compartment of the LDB arena is a variable considered inversely proportional to anxiety-like behaviors. In this study, no difference was observed among the three groups (Figure 2E); therefore, the exercise protocols used in this study were not able to exert an anxiolytic effect in the LDB test.

**Figure 3:**
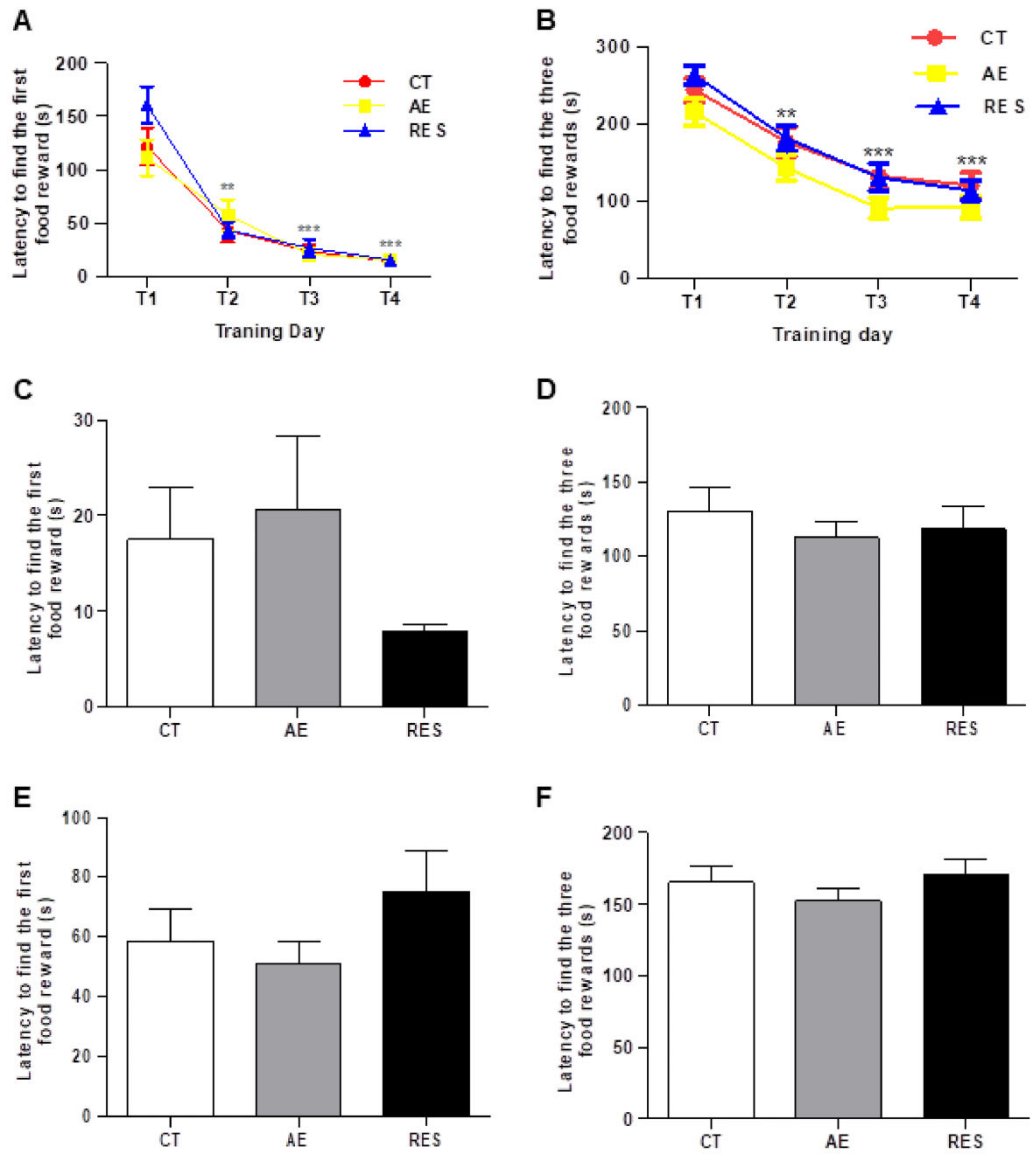
Assessment of cognitive function in the modified hole board test. The learning capacity was measured by the latency to find the first (**A**) and all three (**B**) food rewards during the four training days (T1-T4). **P<0.01, ***P<0.001 compared to day 1 (repeated-measures one-way ANOVA followed by Tukey's *post hoc*). Latencies were analyzed on the fifth day in the location shift phase (**C** and **D**) and in the reversal phase (**E** and **F**) (one-way ANOVA). Data are reported as means±SE (n=14-16 per group). CT: sedentary control; AE: aerobic exercise on the treadmill; RES: resistance exercise on the ladder apparatus.

Regarding the latency to find the first and all three rewards throughout the training phase of the mHB test, one-way repeated-measures ANOVA demonstrated a significant effect of the Time factor (P<0.05) without differences among groups. Shorter latencies were found on the second training day (T2) compared to the first training day (T1) in each group (P<0.01; Figure 3A and B, respectively). Furthermore, on the third and fourth training days (T3 and T4), these latencies continued to be shorter compared to T1 in all groups (P<0.001; Figure 3A and B). The data did not show differences among the groups considering the latency to find the first and the three food rewards, neither in the location shift phase (Figure 3C and D) nor in the reversal phase (Figure 3E and F) of the test. Considering these results, neither treadmill running nor resistance exercise exerted a robust influence on learning ability or cognitive flexibility of healthy animals.

**Figure 4:**
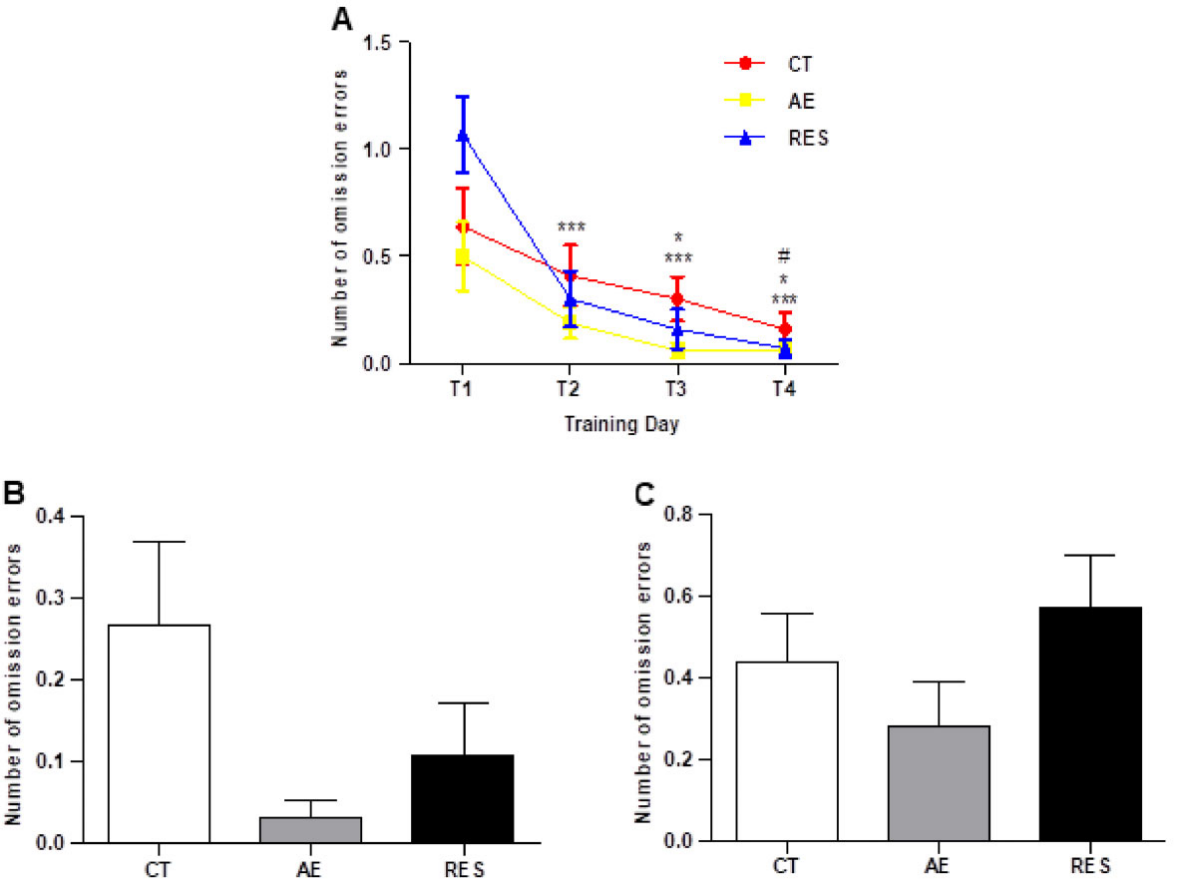
Number of omission errors during the training days (T1-T4) of the modified hole board test (**A**). *P<0.05 compared to the first training day of the AE group; ***P<0.001 compared to the first training day of the RES group; ^#^P≤0.05 compared to the first training day of the CT group (repeated-measures one-way ANOVA followed by Tukey's *post hoc*). The number of omission errors was measured in the location shift phase (**B**) and the reversal phase (**C**) of the task (one-way ANOVA). Data are reported as means±SE (n=14-16 per group). CT: sedentary control; AE: aerobic exercise on the treadmill; RES: resistance exercise on the ladder apparatus.

Throughout the training period of the task, the number of baited bowls not visited (omission errors) was also considered as a parameter inversely associated with learning. Observing the learning curve of each group, the repeated-measures ANOVA demonstrated that all groups presented a reduction in the number of omission errors during training days (Figure 4A), evidenced by the significant effects of the Time factor (F (3,129)=25.28) and the Time × Exercise interaction (F (6,129)=2.33). Moreover, Tukey's *post-hoc* test indicated that this reduction emerged early on the second day compared to the first in the RES group, on the third day compared to the first in the AE group, and less omission errors were observed only on the fourth training day compared to the first in the CT group (Figure 4A). This analysis indicated a mild pro-cognitive effect of both exercise modalities, in which the AE and RES groups showed a more rapid learning than the CT group, and RES presented earlier learning than AE.

Furthermore, no difference was found between the number of omissions in the location shift (Figure 4B) and reversal phases of the task (Figure 4C). Such data suggested that the exercise protocols used in this study did not have an effect on cognitive flexibility in healthy animals on the mHB task.

### Hippocampal plasticity assessment

**Figure 5:**
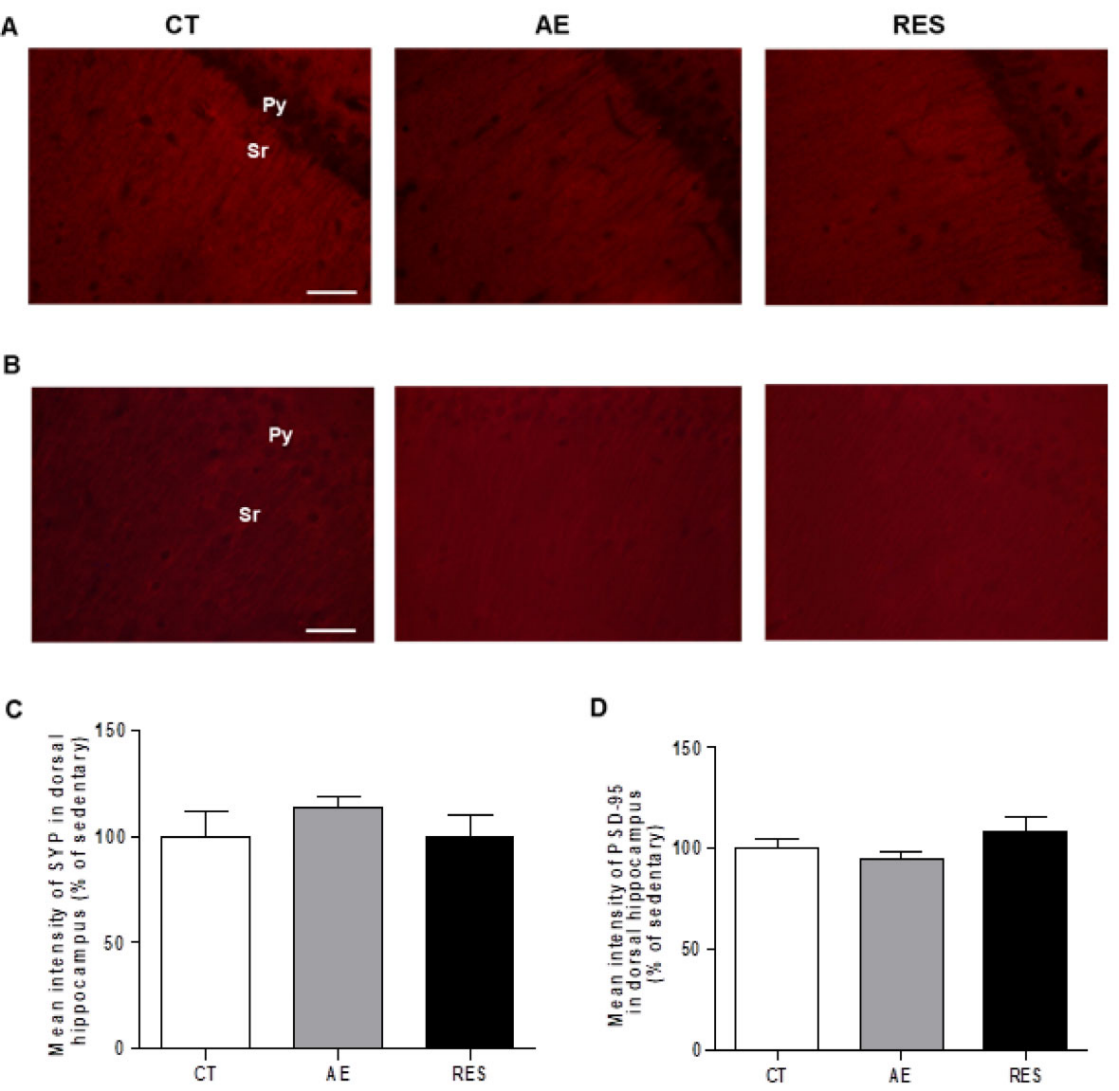
Analysis of neuroplasticity markers in the dorsal hippocampus. Photomicrographs of *stratum radiatum* stained for SYP (**A**) and PSD-95 (**B**) in the dorsal *cornus ammonis 1* region (400× magnification, scale bar=50 μm). Quantification of the immunofluorescence relative intensity (% of sedentary control) for SYP (**C**) and PSD-95 (**D**). One-way ANOVA followed by Tukey's *post hoc*. Data are reported as means±SE (n=4-6 per group). SYP: synaptophysin; PSD-95: postsynaptic density protein 95; Sr: *stratum radiatum*; Py: pyramidal cells layer; CT: sedentary control; AE: aerobic exercise on the treadmill; RES: resistance exercise on the ladder apparatus.

**Figure 6:**
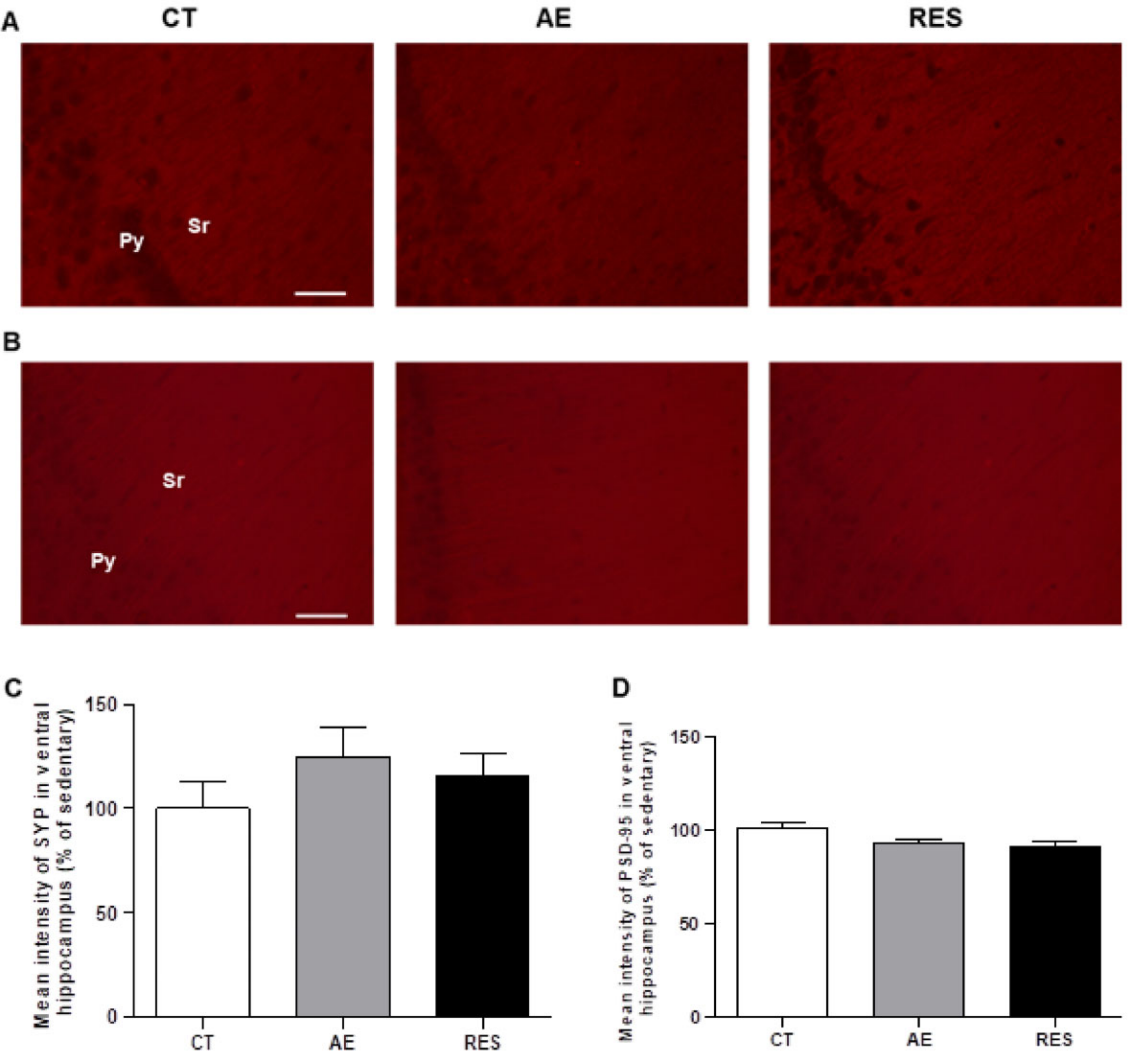
Analysis of neuroplasticity markers in the ventral hippocampus. Photomicrographs of *stratum radiatum* stained for SYP (**A**) and PSD-95 (**B**) in the ventral *cornus ammonis* 1 region (400× magnification, scale bar=50 μm). Quantification of the immunofluorescence relative intensity (% of sedentary control) for SYP (**C**) and PSD-95 (**D**). One-way ANOVA followed by Tukey's *post hoc*. Data are reported as means±SE (n=4-6 per group). SYP: synaptophysin; PSD-95: postsynaptic density protein 95; Sr: *stratum radiatum*; Py: pyramidal cells layer; CT: sedentary control; AE: aerobic exercise on the treadmill; RES: resistance exercise on the ladder apparatus.

One-way ANOVA indicated that neither the aerobic protocol on the treadmill nor the resistance protocol on the ladder apparatus altered the expression of SYP and PSD-95 in the *stratum radiatum* of the CA1 in the dorsal (SYP: F (2,13)=0.69, P=0.518; PSD-95: F (2,11)=1.48, P=0.27) and in the ventral hippocampus of healthy rats (SYP: F (2,10)=0.90, P=0.43; PSD-95: F (2,13)=2.49, P=0.12) (Figures 5 and 6). The results suggested that both exercise modalities performed in this study were not able to modulate such pre- and post-synaptic plasticity markers in hippocampal regions related to anxiety-like behavior (ventral hippocampus) and learning/memory process (dorsal hippocampus).

## Discussion

This study aimed to compare two different exercise interventions - aerobic and resistance training - on emotional/cognitive behaviors and expression of synaptic plasticity markers in the hippocampus of healthy rats. As expected, an anxiolytic effect of the exercise was identified in the elevated plus-maze, particularly in the animals submitted to aerobic training. Contrary to our hypothesis, no significant effect was observed in the anxiety-like behavior of the resistance training group. Additionally, neither exercise protocol had an effect on the cognitive flexibility in the mHB test nor on SYP and PSD-95 expression in the hippocampus.

An anxiolytic effect was observed after aerobic training in the EPM but not in the LDB test. Possibly, the anxiolytic state generated by the aerobic protocol in the EPM was an acute effect of the last training performed on the day before this test. In a similar context, Ahmadalipour and Rashidy-Pour (7) showed that an aerobic protocol caused an increase in the time spent in the light chamber in the LDB on the next day after the last exercise session in healthy animals and animals exposed to morphine during the prenatal period. Nevertheless, when these animals were tested in the EPM, 48 h after the end of the treadmill protocol, only the exposed group had a benefit from exercise. Thus, we can infer that aerobic exercise is able to produce a temporary reduction in state anxiety of healthy animals; a prominent and longer-lasting reduction of anxiety levels can be more apparent if a pathology model is combined with aerobic exercise.

Another point related to the divergence in the results between EPM and LDB analysis could be the performance of both tests in the same animals. As reviewed by Bourin (31), some previous manipulations can substantially alter the rodent's behavior in the LDB test. For example, it was observed that a previous exposition to EPM abolished the anxiolytic effect of diazepam in the LDB (32). Repeated experiences in the LDB also mitigate benzodiazepine effects (33). In this sense, it is possible that the prior exposition to EPM blocked the observation of the anxiolytic effect from aerobic exercise in the LDB test. Both tests are based on ethological features and are widely used in investigations about anxiety-related disorders. Nevertheless, when applied to the same animal, it may affect the results as a bias.

Only the aerobic protocol exhibited an anxiolytic effect, which was an interesting finding. Treadmill running is a forced exercise modality and it elevates the corticosterone levels of animals. However, in a moderate intensity and in chronic protocol, this modality can downregulate the hypothalamic, pituitary, adrenal axis, reducing stress responses and making the rat more resilient to other stressors, such as the EPM apparatus (34,35). By producing a controllable and predictable stress event, resistance exercise was expected to also generate adaptive stress responses, but no emotional effect was observed. Considering recent evidence in humans indicating a positive effect of resistance training on emotional parameters (17), animal studies should be further encouraged in this area. Further studies including models of anxiety-like behavior will help clarify the role of resistance exercise on anxiety state.

Concerning the cognitive evaluation in the mHB test, the decreased latencies to find the rewards and fewer omission errors in the training phase indicated a learning ability. All rats showed reduced latencies to find the first and the three rewards from the second day of the acquisition phase (Figure 3A and B). Moreover, the exercised groups showed faster learning demonstrated by early reduction in the omission errors compared to the control group (Figure 4A). As a pro-cognitive effect of both exercises was observed only on the omission errors, it can be considered a mild effect. Overall, it can be inferred that the treadmill and ladder training done in this study did not substantially modulate animals' performance in the mHB test. Some studies have successfully found cognitive effects of resistance training but frequently adopt an animal model of brain diseases and sometimes use different exercise protocols. For example, in the study of Farzi et al. (36), the authors found improved recognition memory in a rat model of Alzheimer's disease after 8 weeks of resistance training, in which the weight attached to the tail was increased progressively until 100% of the rat weight. In the present study, animals always trained at an intensity of 60% of their maximal physical capacities. Other findings could be identified with different protocols of aerobic and resistance training. A limitation of this study is that the animals were not exercised during the behavioral analysis. Future studies should take into account the maintenance of exercise protocols throughout the functional tests to avoid a possible loss of exercise effects.

Supporting the behavioral results, the physical exercise protocols used in this study did not cause an impact on the pre- and post-synaptic plasticity markers expression (SYP and PSD-95) in the *stratum radiatum* of dorsal or ventral CA1. The total duration and intensity of the exercise protocols can explain this lack of positive effects on cognitive processes and synaptic plasticity. Although the physical capacity of rats was reevaluated weekly and the speed/load was updated, the intensity was maintained at 60%. Aerobic/resistance protocols with progressive intensity, longer daily sessions (30-60 min), and longer total duration (8 weeks) can provide more challenging stimulus to the rodent brain. Such protocols presented positive effects on the spatial and aversive learning and memory tests, and on the hippocampal plasticity markers ki-67 (indicative of cellular proliferation), synaptophysin, synapsin-1, IGF-1, pIGF1-R, BDNF, and Trkβ (10,26,37,38). Furthermore, previous data showed that exercise effects on epigenetic (reduced DMNTs levels) and, probably, on genic transcription can be detected until 1 h after a single exercise session (39). The time-point of sample collection is another aspect that future investigations should pay attention to.

We believe that this is the first study to present a comparative overview of the impact of aerobic and resistance exercise - both at moderate intensity - on anxiety-like behavior and cognitive flexibility in rodents. Although no robust impact of the exercise trainings was identified, the present findings revealed a slight anxiolytic effect after six weeks of aerobic exercise and a mild improvement in learning ability associated with both exercise modalities. No difference was identified for the hippocampal synaptic plasticity indicators evaluated. Future research should consider the potential effects of different protocols of resistance exercise (intensity and duration) more carefully and in diverse conditions. For example, in animals in a pathological situation, as psychological stress, the effect on emotional changes would help clarify the possible impact of resistance exercise.
